# What Promotes Positive Parenting During Breast Cancer? A Cross-Sectional Analysis of Social Support, Emotion Regulation, and Meaning in Life

**DOI:** 10.1007/s12529-023-10196-9

**Published:** 2023-07-06

**Authors:** L. Kelada, O. Zamir

**Affiliations:** 1 Paul Baerwald School of Social Work and Social Welfare, Hebrew University of Jerusalem, Mt. Scopus, 9190501 Jerusalem, Israel; 2 School of Clinical Medicine, UNSW Medicine & Health, UNSW, Sydney, Australia; 3 Kids Cancer Centre, Behavioural Sciences Unit, Sydney Children’s Hospital, Randwick, Australia

**Keywords:** Breast cancer, Parenting, Social support, Meaning in life, Emotion regulation

## Abstract

**Background:**

Motherhood and maintaining the child-parent relationship are top priorities for mothers with breast cancer (BC). The current study aimed to assess the positive intra- and inter-personal resources related to positive parenting among mothers with BC, as these may be promotive factors for positive parenting. Specifically, we examined whether social support (family, friend, spiritual), emotion regulation, and a sense of meaning in life are related to positive parenting among mothers undergoing BC treatments.

**Methods:**

The sample consisted of 100 Israeli mothers (mean age = 46.02 years, SD = 6.06 years) who were undergoing treatment for BC. Participating mothers had at least one child aged 6–17 years. Participants were recruited via closed social media groups to complete a questionnaire containing: the Alabama Parenting Questionnaire, Cancer Perceived Agents of Social Support tool, Emotion Regulation Questionnaire, Meaning in Life Questionnaire, European Organization for Research and Treatment of Cancer Quality of Life Questionnaire (EORTC QLQ-C30), and sociodemographic and clinical questions. We used structural equation modeling to regress the study variables on positive parenting.

**Results:**

Friend support (β = .35, *p* = .009) and meaning in life (β = .30, *p* = .012) were significantly related to positive parenting. Family support, spiritual support, cognitive reappraisal, and the sociodemographic and clinical variables were not related to positive parenting.

**Conclusions:**

Our study suggests that promoting meaning in life and friend support may be key to helping mothers to sustain positive parenting behaviors throughout their cancer treatment. Future research may examine whether psychosocial interventions which foster meaning in life and friend support impact positive parenting among mothers with BC.

**Supplementary Information:**

The online version contains supplementary material available at 10.1007/s12529-023-10196-9.

## Introduction

Breast cancer (BC) is the most commonly diagnosed cancer in women, with an estimated 2.3 million new cases each year [1]. The median age for a BC diagnosis is 62 years, but women under 50 years of age can also be at risk of BC [2]. At a younger age, women often have at least one dependent child at home at the time of diagnosis [3]. Hence, while coping with BC treatments and their physical side effects (e.g., pain, fatigue) and adverse psychological effects (e.g., depression) [4], mothers need to maintain their parental role. The physical and psychological challenges faced by mothers going through cancer treatments may affect their parenting [5–7]. Having BC predicts lower maternal satisfaction and self-efficacy [8]. Moreover, greater psychological distress during BC treatments is associated with the use of more harsh and inconsistent discipline [9].

There is variability — between and within mothers — in how BC affects them and their parenting. For instance, research has shown that some mothers perceive themselves to be unable to fulfill their parenting role after a BC diagnosis, which is related to guilt, sadness, frustration, depression, and stress [6, 10, 11]. Other studies, however, have found that mothers may become more involved with their children after diagnosis [7, 12, 13] and may experience improvements in their relationship with their children [6, 14, 15]. Still, not much is known about promotive factors (i.e., factors that directly predict positive outcomes) [16] for positive parenting in mothers with BC [10]. Given that positive parenting is a major protective factor for child resilience in the face of adversity [17], this study aimed to identify promotive resources for positive parenting practices when women are facing BC treatments.

### Positive Parenting Practices

Parenting is a major concern for mothers with BC, who often experience high worry about their children and fears of “leaving them behind” [6]. Research spanning over four decades has recognized parenting as a central protective factor for healthy child development when families cope with severe life events [17]. Positive parenting practices refer to a parent’s continual effort to meet the needs of their children and include caring, teaching, leading, communicating, and providing [18]. In this study, we focused on two main positive parenting practices: (a) positive reinforcement and (b) parental involvement including emotional support, care, and communication [19]. Effective parenting behaviours such as these facilitate adaptive psychological and behavioural development of children [20]. Positive parenting may protect children against developing antisocial behavior and may promote the acquisition of prosocial behavior [21–23].

### The ABC-X Model of Family Adjustment and Adaptation

Parent illness — and in particular the stress associated with it — may prohibit positive parenting [24, 25]. However, promotive factors may facilitate better parental functioning [16]. The ABC-X model posits a theoretical foundation on the mechanism by which promotive factors may foster positive parenting practices in the context of stress. This model was originally developed by Hill [26] to explain a family’s response to stressors, ending with the conclusion of the crisis, and was later expanded to the double ABC-X model to explain the adaptation of a family to an ongoing stressor, including postcrisis coping processes over time. The model postulates that the adaptation of family members to stress is a function of three factors, including (aA) the magnitude of a stressor, (bB) the resources available to the family, and (cC) the extent to which the family perceives the stressor as positive or manageable. All three factors are interrelated and jointly lead to the adaptation of a family to a crisis (xX) [27–29]. The ABC-X and double ABC-X models have a wealth of empirical support in several fields including family illness and disability [30–32].

BC is a life-threatening disease that may cause major stress for mothers [33]. The double ABC-X posits that stress may pile up over time [29]. Indeed, dealing with cancer may lead to accumulative long-term stressors. For example, the treatments for BC typically involve surgery and radiation therapy, systemic therapies such as endocrine (hormone) therapy, chemotherapy, or targeted biologic therapy [34]. These treatments, which often include severe side effects (e.g., pain, fatigue, postmenopausal symptoms), may adversely affect the psychological well-being and quality of life of mothers [for a review see 4].

Despite the stressors associated with the disease severity and treatments, both the ABC-X and the double ABC-X models posit, that positive intra- and inter-personal resources which mothers can draw upon, as well as positive perceptions of their recovery experiences, may facilitate family resilience. Hence, we examined whether intra-and inter-personal recourses (i.e., emotion regulation, social support) and perception, namely a sense of meaning in life, promote positive parenting practices when facing cancer treatments.

### Social Support

Social support includes emotional (e.g., provision of care, love, and empathy), instrumental (provision of tangible assistance), cognitive/appraisal (provision of affirmation), and informational support (provision of information to aid decision-making) [35]. Social support is a vital resource when parents are dealing with adversity that can promote positive parenting [36, 37]. Social support is postulated to reduce the effects of stress through supportive actions (e.g., advice, reassurance), which enhance coping performance and help the individual to appraise adverse events as less stressful [38, 39]. The social network, social embeddedness, and social climate precede social support, which in turn affects a range of personal outcomes including mental health and coping [35].

Research has consistently found that mothers with BC strive to maintain normal daily routines for the sake of their children [40]. Mothers may, therefore, lean on their social support to achieve this and to receive support in their parental role [10, 40]. Specifically, research has shown that instrumental or practical support is commonly available to mothers (e.g., childcare) but that it can be difficult to trust others to care for their child/ren [6, 41]. Moreover, mothers often report emotional support to be an unmet need during their cancer treatment [6]. Providers of social support vary significantly between studies, with research showing that partners, family members, local religious/spiritual groups, and local cancer support networks can all be important sources of support [3, 40, 41]. However, the unique contribution of each of these support sources to positive parenting practices of mothers undergoing BC treatments is unclear.

### Emotion Regulation

Emotion regulation refers to “the processes by which individuals influence which emotions they have, when they have them, and how they experience and express these emotions” [42]. Emotion regulation has been found to play a significant role in women’s adaption to a BC diagnosis [3, 43, 44]. Gross and John [45] argue that there are two types of emotion regulation strategies. Emotional suppression involves regulating emotions by suppressing them and may lead to adverse psychosocial outcomes [46]. Cognitive reappraisal involves reframing the way one thinks about a stressor and is consistently linked with positive psychosocial outcomes [46]. Because we were interested in studying promotive factors, we focused on cognitive reappraisal as a personal resource for positive parenting.

Cognitive emotion regulation, such as reappraisal, is particularly important for parental functioning in stressful situations, allowing them to remain calm, cope with stress and respond to their children in a more positive manner [47]. Emotion regulation is associated with positive parenting practices, including sensitive, involved parenting [47, 48]. Furthermore, cognitive reappraisal, but not emotional suppression, has been shown to be linked with lower parenting stress among mothers with BC [49]. Interestingly, cognitive reappraisal was not related to parenting stress among mothers without BC [49], suggesting that cognitive reappraisal may be particularly important intrapersonal resource for mothers with BC. Further research is needed to examine whether cognitive reappraisal is a promotive factor for positive parenting among women with BC [10].

### Meaning in Life

Meaning in life is a multi-faceted concept that encompasses the purpose and value in life, the attainment of life goals, and a subsequent sense of fulfillment [50, 51]. Meaning in life can also encompass spirituality and religiosity for some but is not a necessary element to attain meaning [51]. When facing severe stressors such as cancer, it often violates core beliefs about the world as predictable and fair, which may elicit the search for meaning to rebuild the meaning systems [52]. The process of searching for meaning involves attempts to make sense of adversity, [53, 54], and it may result in finding meaning. A sense of meaning includes the modification of basic beliefs about the world as manageable, benevolent, and meaningful, and as such perceived as less threatening [52]. Whereas the search for meaning may tax the psychological well-being [53, 54], the presence of meaning in life is associated with positive psychological adjustment among women with BC [55, 56]. We, therefore, focused on the presence of meaning as a promotive factor for positive parenting.

Finding life as meaningful may facilitate better parental functioning. For example, the presence of meaning was associated with a lower level of parenting stress, whereas search for meaning was associated with higher stress among new parents before and during the COVID-19 pandemic [57]. As mothers are confronted with the threat of leaving their children orphans [13], finding meaning is common among mothers with cancer even more than among nonmothers with BC [56]. Part of changing the meaning of life may include shifting maternal behaviors, such as being more available and positively involved with children. It may also shape their meaning in life around their role of “motherhood” above all else [13]. However, whether meaning in life is related to better positive parenting outcomes remains unknown.

### The Current Study

Motherhood and maintaining the child-parent relationship are top priorities for mothers with cancer [6]. Some mothers with BC describe their role of motherhood as being the most important part of life, more so than the experience of cancer [7]. As such, it is important to examine the intra- and inter-personal resources related to positive parenting during BC treatment, as these may be promotive factors for positive parenting. In this study, we examined whether social support (partner and family support, friend support, spiritual support), emotion regulation, and a sense of meaning in life are related to positive parenting among mothers going through BC treatments. We hypothesize that greater social support, provided by either family, friend, or spiritual sources, emotion regulation, and a sense of meaning in life, will all be associated with a greater level of positive parenting among mothers dealing with BC treatments.

## Method

### Participants

The sample was comprised 100 Israeli mothers, all of whom reported being diagnosed with BC and undergoing BC treatments. Participating mothers had at least one child aged 6–17 years. This age group has been identified as at-risk for emotional and behavioural problems when their parent is diagnosed with cancer [for a review, see 58]. The mean age of mothers was 46.02 (*SD* = 6.06), and most of them were married (88%). Other mothers reported being cohabiting with a partner (4%), involved in a relationship but not cohabiting (1%) or divorced (7%). Of those who were married or in a relationship, the relationships had lasted for an average of 17.58 years (*SD* = 6.23). Participants reported having one to seven children (*M* = 2.94, *SD* = 1.01). Two-thirds of participants had earned at least a Bachelor’s degree (66%) and reported an average of 15 years of education (*SD* = 2.75). Most of the mothers worked at least part-time (68.7%), were born in Israel (90%), and considered themselves secular (67%) and Jewish (97%). The monthly family income varied between 0–10,000 ILS (19.6%), 10,000–25,000 ILS (61.8%), and 25,000 or higher (18.6%). It should be noted that the average family income in Israel in 2018 was 24,872 ILS ($7765) per month [59].

The majority of the mothers reported being diagnosed within the past 12 months (66%). Disease stages were zero (2.1%), one (13.8%), two (37.2%), three (27.7%), or four (19.2%). Mothers reported they were currently receiving cancer treatments, including chemotherapy (40%), biological therapy (37%), hormonal therapy (35%), radiation (25%), or other treatments (6%).

We performed an a priori power analysis using G*Power Version 3.1.9.7 [60] to determine the necessary sample size for detecting a medium effect size (*β* = 0.30) with 80% power, given eight predictors. The analysis revealed that a sample size of 82 was required.

### Procedure

Following an IRB approval from Hebrew University, School of Social Work Ethics Committee, we recruited participants between July 2018 and September 2019 using a convenience sampling method. Advertisements of the study were published in closed online BC groups on social media. First, participants were invited to fill out a short, online screening questionnaire to assess their compatibility with the research inclusion criteria, which included being a native Hebrew speaker, mother to children aged 6–17 years, and diagnosed with BC and undergoing cancer treatments at the time of the study. Mothers who met the inclusion criteria were automatically referred to an online survey where they were asked to complete an informed consent form followed by an anonymous online self-report questionnaire. Out of the 243 women who were enrolled in the study, 121 were eligible and consented to participate (49%). However, 21 responses (17%) were removed due to failing quality checks, resulting in a final sample of 100 women.

### Measures

#### Clinical Characteristics

Participants completed the Hebrew version of the European Organization for Research and Treatment of Cancer Quality of Life Questionnaire (EORTC QLQ-C30) [61], from which we used the two-item global health status scale. Respondents rated their overall health and quality of life over the previous week on a scale from 1 “very poor” to 7 “excellent.” An average score was computed followed by a linear transformation. Scores ranged between 0 and 100, with higher scores indicating higher functioning. For more details on the scoring procedures, see the EORTC QLQ-C30 Scoring Manual [62]. The EORTC QLQ-C30 is a validated and widely used measure of quality of life in cancer patients across various diagnoses and cultures [61, 63, 64]. The measure has shown good internal consistency [61, 65, 66], including in the current study (*α* = 0.92).

#### Parenting

The Alabama Parenting Questionnaire [APQ; 67] includes 42 items assessing five domains of parenting practices for parents of children aged 6–17 years. In this study, we used two subscales assessing positive parenting practices, including the 6-item positive reinforcement subscale (e.g., “You compliment your child when he/she does something well.”) and the 10-item parental involvement subscale (e.g., “You have a friendly talk with your child”). Respondents indicated how frequently they enact each behavior from 1 “never” to 5 “always.” A total sum score was computed, with higher scores indicating more positive parenting practices. The APQ has demonstrated good convergent validity, divergent validity, and good factor structure [67, 68]. The positive parenting subscales of the APQ had good internal reliability with the current sample (Cronbach’s *α* = 0.86).

#### Social Support

The Cancer Perceived Agents of Social Support tool [CPASS; 69] is a 12-item self-report measure assessing four sources of support (family, spouse, friends, and spiritual) for cancer patients. Participants were asked to report the level of support received from each source in three domains (psychological, instrumental, and cognitive) and rated the support on a scale ranging from 1 “not at all” to 5 “a lot.” A mean score was computed with higher scores indicating greater perceived support. The questionnaire has been found to have good content validity, convergent validity, structural validity, and predictive validity among BC patients and has shown good internal reliability in previous studies in Israel (*α* = 0.78–0.93) [70], as well as in this study (Cronbach’s *α* = 0.88–0.95). Family and spouse support were found to have high multicollinearity in this study, so only family support was included in the analyses.

#### Emotion Regulation

The 6-item cognitive reappraisal subscale was used from the Emotion Regulation Questionnaire (ERQ) [45]. This scale assesses participants’ use of cognitive reappraisal strategies to regulate their emotions (e.g. “When I’m faced with a stressful situation, I make myself think about it in a way that helps me stay calm”). Participants rated their level of agreement with each statement from 1 “strongly disagree” to 7 “strongly agree,” and scores were then averaged across the items. Higher scores indicate greater cognitive reappraisal. The ERQ has been shown to have high internal reliability, high test–retest reliability, and acceptable convergent and discriminant validity [45] and had good internal reliability with the current sample (Cronbach’s *α* = 0.71).

#### Meaning in Life

Meaning in life was measured using the Meaning in Life Questionnaire (MLQ) [71]. The MLQ contains two subscales: the presence of meaning and the search for meaning. In the current study, we use the presence of meaning subscale which consists of five items (e.g., “My life has a clear sense of purpose”). Respondents indicated their level of agreement with each item on a 7-point scale from 1 “absolutely true” to 7 “absolutely untrue,” and scores were averaged across the items. Higher mean scores indicate a greater sense of meaning in life. The MLQ demonstrates good convergent validity, discriminant validity, and factor structure [71]. The presence of meaning subscale of the MLQ had good internal reliability with the current sample (Cronbach’s *α* = 0.84).

#### Socio-demographic Characteristics

Participants reported their socio-demographic characteristics, including their age, country of birth, marital status, economic status, education, religious affiliation, and health status.

#### Disease and Treatment

Participants reported whether they were diagnosed with BC (0 = *No*, 1 = *Yes*), the stage of the disease (0–4), the time since diagnosis (1 = *up to 12 months*, 2 = *more than a year*), and whether they were undergoing cancer treatment, including chemotherapy, biological therapy, hormonal therapy, radiation, or other treatments.

### Data analysis

First, we used IBM SPSS Version 25 to produce descriptive statistics, Pearson’s correlations between the continuous study variables, an independent samples *t*-test to assess differences in positive parenting according to marital status, and an ANOVA to assess differences in positive parenting according to income. Missing values occurred across variables and participants, but Little’s Missing Completely at Random (MCAR) test indicated that the data were missing at random [χ^2^(23) = 14.53, *p* = 0.91]. Therefore, using IBM Amos Version 25, the model was tested using full information maximum likelihood (FIML) estimation, which uses all available information from the observed data to generate parameter estimates [72]. The model was saturated. We specified a path model using structural equation modeling (SEM) via IBM Amos 25. In this model family support, friend support, spiritual support, cognitive reappraisal, and meaning in life were regressed on positive parenting. Because cancer-related stressors can affect maternal functioning [27–29], we controlled for disease stage, time from diagnosis, and quality of life.

## Results

Descriptive statistics and zero-order Pearson’s correlations for the study variables are presented in Table 1. Positive parenting practices were positively correlated with friend support (*r* = 0.34, *p* = 0.002) and meaning in life (*r* = 0.31, *p* = 0.004). We also noted several other significant correlations: (i) meaning in life was significantly related to family, friend, and spiritual support; (ii) family and friend support were highly, positively correlated; and (iii) family and friend support were negatively correlated with time since diagnosis (Table 1).

**Table 1:** Means, standard deviations, and zero-order correlations of the study variables

	*M*(*SD*)	Positive parenting	Time since diagnosis	Global health status	Family support	Friend support	Spiritual support	Cognitive reappraisal	Meaning in life
Positive parenting	64.69 (8.17)	1							
Time since diagnosis	9.48 months (3.40 months)	− .083	1						
Global health status	72.92 (15.19)	.038	− .138	1					
Family support	3.51 (1.10)	.210	− .314^**^	.165	1				
Friend support	3.59 (1.04)	.338^**^	− .247^*^	.076	.602^***^	1			
Spiritual support	2.42 (1.55)	.124	.152	− .127	.092	.322^**^	1		
Cognitive reappraisal	30.11 (6.24)	.009	− .049	− .034	.034	.043	− .011	1	
Meaning in life	4.86 (1.17)	.312^**^	− .059	.010	.451^**^	.360^**^	.251^*^	.200	1

^*^p < .05; **p < .01; ***p < .001

Positive parenting was not significantly correlated with age (*r* = − 0.03, *p* = 0.77), number of children (*r* = − 0.18, *p* = 0.09), and education level (*r* = − 0.08, *p* = 0.44). Positive parenting also did not significantly differ according to marital status (*t* (89) = 0.16, *p* = 0.874) and income (*F* (2, 86) = 0.70, *p* = 0.932). Therefore, we did not include these demographic variables in our model.

Results from the SEM are shown in Table 2. Friend support (*β* = 0.35, *p* = 0.009) and meaning in life (*β* = 0.30, *p* = 0.012) were significantly related to positive parenting practices. Family support, spiritual support, and cognitive reappraisal were not related to positive parenting. The clinical characteristics were also not related to positive parenting. In total, the model explained 19.7% of the variance of positive parenting practices.

**Table 2:** SEM analysis for positive parenting

	B	*SE*	*p* value
*Clinical characteristics*			
Time since diagnosis	2.292	2.005	.253
Stage	− .860	.908	.343
Global health status	− .074	.056	.186
*Positive interpersonal resources*			
Family support	− .478	.994	.630
** Friend support**	**2.760**	**1.054**	**.009**
Spiritual support	− .329	.585	.573
*Positive intrapersonal resources*			
Cognitive reappraisal	− .058	.130	.659
** Meaning in life**	**2.053**	**.814**	**.012**

Significant paths are bold

## Discussion

The current study aimed to assess the positive intra- and inter-personal resources related to positive parenting among mothers with BC. Our findings partially support our hypotheses. Specifically, two promotive factors were found to be associated with positive parenting: greater friend support and meaning in life. The results of the study are in line with the ABC-X and the Double ABC-X models which postulate that despite exposure to stressors (i.e., BC severity and disrupted well-being), which can accumulate over time (time since diagnosis), having greater resources (i.e., friend’s support), and a more adaptive perception of the stress and coping process (i.e., meaning in life) can facilitate better adjustment of family members to adversity. Importantly, friend support and meaning in life are related to positive parenting, above and beyond several stressors, including the disease severity, the quality of life following the treatments, and the time since diagnosis.

The results of the study broaden our understanding of what promotes resilience among mothers dealing with BC treatments in terms of maintaining positive parenting. The extant research on the maternal functioning of mothers with BC has mostly focused on parental tasks related to the disease, such as communicating about cancer [6]. Given that parenting is a major protective factor for child development in the face of adversity [17], more research in needed on this topic.

Consistent with our hypothesis, friend support was related to positive parenting. The findings of the study are consistent with the theory of stress and coping which postulates that support allows individuals to cope better when encountering threats and uncertainty [38]. When women go through BC, their friends may provide emotional support, attend appointments, or be a source for women to confide in and engage in shared interests [73]. To a lesser extent, friends may also provide practical support. Some friends may be survivors of BC themselves and thus able to provide a unique level of support [73]. The support of friends may be particularly important during treatment for BC, as it fosters higher post-traumatic growth [74].

Although previous research has found that family support or spiritual support are linked with lower psychological distress among mothers with BC [3, 40, 41], in the present study, neither family nor spiritual support was found to be related to positive parenting practices. As such, the support of friends may be a unique promotive factor when it comes to positive parenting practices for mothers with BC. Under stressful conditions, women may utilize the “tend and befriend” strategy (i.e., allowing themselves to be assisted by their social network, particularly women friends) to ensure the protection of their offspring [75]. Moreover, friends of a similar parenting age may provide parenting strategies or support that is in-line with the diagnosed mother’s perspectives on parenting. Still, research has shown that some friends may be unable or unwilling to provide the type or level of support needed by a mother with BC, with some studies showing that women may isolate themselves from their friends [76]. Also, mothers with BC commonly report not receiving adequate emotional support from their social network [6]. Furthermore, although greater support from friends was found to be a promotive factor for positive parenting during BC treatments, the current study did not assess to what extent this support met the mothers’ needs and how satisfied they were with the support they received. Therefore, future research should assess which type of support provided by friends, and mothers’ subjective evaluation of this support, and how this relates to their parenting during BC treatment.

Meaning in life was also found to be related to positive parenting. Meaning in life is a significant protective factor in life-threatening conditions allowing traumatized individuals to reconstruct basic beliefs concerning their secure existence in the world. Research has found that meaning making may help individuals to overcome and even grow in the face of trauma [77]. Further, finding meaning has shown to benefit parental stress in the transition to parenthood [57]. Our research expands the scope of the extant knowledge by illuminating the promotive role of meaning in life for positive parenting practices of mothers undergoing BC treatments. Psychological distress associated with stressful conditions including BC can impair parental functioning [5–7]. In contrast, meaning in life supports positive psychological adjustment in women with BC [55, 56]. As such it could be that finding meaning in life is a protective factor against psychological distress and thereby promotes positive parenting. In addition, finding new meaning in life may involve a change in their maternal role including prioritizing motherhood above all else and being more available and positively involved with children [13].

The tendency to use cognitive reappraisal to regulate emotions was not associated with positive parenting. Prior research has shown that cognitive reappraisal is associated with lower parenting stress in mothers with BC [49], indicating that cognitive reappraisal protects against negative parenting experiences. However, our study showed that cognitive reappraisal may not be a promotive factor for positive parenting in mothers with BC. It could be that dealing with a life-threatening disease may require other resources to preserve positive parenting [33]. For example, while cognitive reappraisal is an effective strategy for dealing with general stress and distress [46], using coping strategies such as meaning in life may be more effective in managing the emotional distress associated with a life-threatening illness. The ability to deploy an appropriate strategy to manage intense emotional distress may help mothers to engage in positive interactions with their children. Still, further research is needed to confirm our findings.

Finally, we also observed several significant bivariate correlations which were not central to our research aim, but which provide further context to the main findings. First, family support, friend support, and spiritual support were all correlated with meaning in life which suggests that support may contribute to meaning in life which in turn contributes to positive parenting. Moreover, the positive correlation between spiritual support and meaning in life is consistent with Jim and colleagues’ [51] conclusion that meaning in life may encompass spirituality for some individuals. Second, family support and friend support were negatively correlated with time since diagnosis, with support reducing over time. This may correspond with caregiver fatigue across the illness trajectory [78] and aligns with the Double ABC-X model that resources diminish over time [29]. It may be important for future interventions to work to extend support, especially friend support, into late stages of treatment and post treatment to maintain positive parenting protective factors.

### Strengths and Limitations

For several decades, researchers and clinicians have called for a greater emphasis on positive outcomes, alongside negative outcomes when examining physical well-being [79, 80]. Our study helps address this gap in the literature by focusing on promotive factors for positive parenting among mothers with BC. Nevertheless, our study also had limitations. The cross-sectional design restricts the interpretation of the study, such that causality cannot be inferred. In addition, the use of self-report measures could be subject to several biases (e.g., self-presentation) and statistical artifacts (e.g., common method variance). Future studies should use multi-informant and multi method designs (e.g., observations). Lastly, the study sample was relatively small and homogenous (i.e., included 97% Israeli Jewish mothers, most of whom were married and educated to the level of a Bachelor’s degree), which limits the generalizability of our findings. Replication of the model in culturally diverse populations is therefore needed.

### Clinical Implications

Maintaining their role as mothers is extremely important to women undergoing BC treatment with young children at home. Indeed, motherhood may be an important factor which helps women cope adaptively to their illness, as they feel motivated to survive and cope for the sake of their children [10, 13]. As such, it is imperative to develop effective interventions to support mothers with BC continue to practice positive parenting. Our study suggests that promoting meaning in life and friend support may be key to helping mothers to sustain positive parenting behaviors throughout their cancer treatment.

Evidence shows that interventions which bolster meaning in life can improve women’s well-being after a BC diagnosis [81–83]. For example, Sun et al. [82] developed a 12-week logotherapy intervention for women with BC and gynecological cancers designed to help them find meaning in their lives (see Wong [84] for a discussion on logotherapy). The authors found that the intervention was able to reduce depression and demoralization among the women [82]. The current study shows that such interventions which target meaning in life may also help to facilitate positive parenting for mothers of young children. Future research would benefit by assessing the effect of these interventions on positive parenting among mothers with BC.

The current study found a strong and positive relationship between friend support and positive parenting. However, friends may not always be aware of the best ways to support their friend with BC [76] and may feel uncertain about their role. Interventions which aim to empower friends and reduce their uncertainty may help increase their supportive behaviors. Social workers may be ideally placed to help empower friend support. Future research should assess this and examine whether increasing support from friends can also increase positive parenting among mothers with BC. Furthermore, peer support between women undergoing treatment for BC may also be an important tenant in perceived friend support. Social workers and community organizations may facilitate support between mothers of young children while undergoing treatment for BC.

## Conclusion

It is extremely important to mothers of young children that they are able to maintain positive parenting practices while undergoing BC treatment. The current study found that support from friends and having meaning in life may be factors which can promote positive parenting during cancer treatment. Important avenues for future research include examining whether psychosocial interventions which foster a sense of meaning in life and friend support have an impact positive parenting among mothers with BC.

### Supplementary Information

Below is the link to the electronic supplementary material.

Supplementary file1 (DOCX 43 kb)

## Data Availability

The data that support the findings of this study are not publicly available due to privacy or ethical restrictions, and the full dataset is not able to be released due to ethical restrictions. Requests may be made to the authors.
