# Supplementary material for: What Promotes Positive Parenting During Breast Cancer? A Cross-Sectional Analysis of Social Support, Emotion Regulation, and Meaning in Life
Source: Int J Behav Med. 2023 Jul 6;31(4):595–604. doi: 10.1007/s12529-023-10196-9 (PMC11269428; doi:10.1007/s12529-023-10196-9)
Supplement: Supplementary file 1 — Supplementary file1 (DOCX 43 kb) [file 12529_2023_10196_MOESM1_ESM.docx]

*Figure. 1*

*SEM Analysis for Positive Parenting*

Meaning In Life

B =2.053 *

Psitive Parenting

Cancer Stage

Spritual Support

Family Support

Cognitive Reaprisal

B =-.058

B =-.478

B =2.760**

Friend Supprt

*R*^2^= .197

Time since diagnosis

Global Quality Of Life

B =-.329

B =-.074

B =-.86

B =2.292

*Note*. Significant paths are bold. * *p* < .05. ** *p* < .01
